# A pilot randomised controlled trial of befriending by volunteers in people with intellectual disability and depressive symptoms

**DOI:** 10.1111/jir.12886

**Published:** 2021-09-27

**Authors:** A. Ali, E. McKenzie, A. Hassiotis, S. Priebe, B. Lloyd‐Evans, R. Jones, M. Panca, R. Omar, S. Finning, S. Moore, C. Roe, M. King

**Affiliations:** ^1^ Epidemiology and Applied Clinical Research Department, Division of Psychiatry University College London London UK; ^2^ Research and Development Office Goodmayes Hospital, North East London NHS Foundation Trust London UK; ^3^ Unit for Social and Community Psychiatry, WHO Collaborating Centre for Mental Health Services Development Queen Mary University of London London UK; ^4^ Priment Clinical Trials Unit University College London London UK; ^5^ Department of Statistical Science University College London London UK; ^6^ Outward Housing Outward, Newlon House London UK; ^7^ The Befriending Scheme Sudbury Suffolk UK

**Keywords:** Befriending, Depressive symptoms, Intellectual disability, Pilot study, Randomised controlled trial, Volunteers

## Abstract

**Background:**

People with intellectual disability (ID) are more likely to experience chronic depression compared with the general population, which may be compounded by loneliness and lower levels of social support. Befriending aims to provide social support and promote engagement in community activities. No randomised controlled trials have examined whether befriending improves symptoms of depression and social outcomes in people with ID. The aim of this pilot trial was to assess the feasibility and acceptability of a future larger trial of one‐to‐one befriending by volunteers in people with ID and depressive symptoms.

**Methods:**

Participants were adults with mild or moderate ID with a score of 5 or more on the Glasgow Depression Scale for People with Learning Disabilities (GDS‐LD). They were randomised to the intervention arm (matched to a volunteer befriender for 6 months) or the control arm (usual care). Volunteers received training and supervision provided by two community befriending schemes. The main outcomes were feasibility of recruitment (minimum target *n* = 35), retention rate of participants, adherence (minimum 10 meetings), acceptability of the intervention, changes in depressive symptoms (assessed at baseline and 6 months) and feasibility of collecting data for a health economic analysis.

**Results:**

Recruitment was challenging, and only 16 participants with ID and 10 volunteers were recruited. Six participants were matched with a volunteer and no participants dropped out (except for two volunteers). Four participants completed 10 meetings (mean 11.8). Befriending was thought to be acceptable, but modifications were suggested. An exploratory analysis suggested that GDS‐LD score was lower in the intervention group compared with the control group after adjusting for baseline scores, but not significant (adjusted mean difference: −4.0; 95% confidence interval: −11.2 to 3.2).

**Conclusions:**

A large trial would not be feasible based on the recruitment strategies employed in this study. A further feasibility study addressing these challenges or the use of other study designs should be considered.

## Introduction

Chronic depression is more prevalent in individuals with intellectual disability (ID) (Richards *et al*. 2001) compared with the general population. Exposure to multiple social disadvantages that include stigma (Emerson 2013; Ali *et al*. 2015), loneliness and low social support (Ailey *et al*. 2006) may contribute to the chronicity of depression in this population.

One evidence‐based approach to treating depression is behavioural activation, which aims to increase engagement in activities that provide pleasure and meaning. There is evidence that behavioural activation is effective in reducing symptoms of depression in people with ID (Jahoda *et al*. 2017). Encouraging people with ID and depressive symptoms to engage in community activities can be particularly challenging, not only because of low motivation associated with depressed mood, but due to difficulties accessing the community resulting from lack of skills or confidence. Befrienders may therefore play a crucial role in supporting behavioural activation through promoting community engagement.

Befriending, defined as a friendship‐like relationship between two or more individuals that is initiated, supported and monitored by an external agency (Dean and Goodlad 1998), varies widely in practice (Thompson *et al*. 2016) but aims to enhance social and emotional support and community participation. There is some evidence that befriending may reduce depressive symptoms in the general population (Mead *et al*. 2010) but evidence in people with ID is limited. A matched comparison study of mentoring (Stancliffe *et al*. 2015), which aimed to increase participation of older adults with ID in community groups, found that mentoring led to significantly improved social satisfaction and there was a trend towards lower symptoms of depression on the carer reported version of the Glasgow Depression Scale for People with Learning Disability (GDS‐LD; Cuthill *et al*. 2003). There have been no published randomised controlled trials of befriending in people with ID.

In this pilot randomised controlled trial, we examined the feasibility, adherence, safety (adverse events) and acceptability of befriending, delivered by lay volunteers and monitored by community befriending services. In addition, we explored changes in depressive symptoms and social outcomes (self‐esteem, loneliness, quality of life, social support and social participation) in individuals with ID, as well as social outcomes in volunteers (wellbeing, self‐esteem, loneliness and attitudes towards people with ID). The feasibility of collecting data on service use, costs and health‐related quality of life for a health economic evaluation, was also explored.

## Methods

A published protocol for the study (Ali *et al*. 2020) provides details about the planned procedures, methodology, analysis and conduct of the study. Participants with ID were randomised using a web‐based randomisation system (Sealed Envelope) to either the befriending intervention arm or an active control arm. Both groups received a booklet of local activities and resources and had access to their usual care. Our aim was to approach 50 eligible people with ID with a view to recruiting 40 participants. We had planned to conduct follow‐up assessments at 6 and 12 months post‐randomisation. However, due to delays in setting up the study, only the assessment at 6 months (post‐intervention) was completed. The research assistant who completed the follow‐up assessment was blind to group allocation.

As part of the process evaluation, semi‐structured interviews were conducted with participants with ID, volunteers, volunteer coordinators and carers in order to obtain their feedback about their experiences of the intervention and study processes, whether the intervention was perceived to be beneficial and what aspects could be improved further. Interviews were audio‐recorded, transcribed and analysed using NVIVO by two researchers. Thematic analysis was used to code the data and to identify relevant themes.

### Recruitment

Ethical approval was received from the London‐City and East Research Ethics Committee (reference 18/LO/2188) in January 2019. Participants with ID were recruited from referrals to two community befriending services (based in Suffolk and Hackney) and from four community ID services at the North East London NHS Foundation Trust (NELFT) between April 2019 and October 2019. Clinicians identified potentially eligible participants from their caseload and made the initial approach to discuss the study and provided an accessible information sheet. If the potential participant was interested in taking part, their details were passed to the research assistant, who contacted the participant, assessed their eligibility and obtained informed consent prior to completing the baseline assessment.

Volunteers were recruited by the befriending services through advertisements in local newspapers, social media, websites and via recruitment campaigns at higher education colleges. An informal interview was conducted to assess their suitability and informed consent was obtained prior to enrolling into the study. All volunteers completed relevant training (face to face and e‐learning) before being matched to a participant.

### Inclusion and exclusion criteria

Participants with ID were included if they were aged 18 or over; had mild or moderate ID (IQ of 35–69), assessed using the Wechsler Abbreviated Scale of Intelligence (WASI‐II; Wechsler 2011); were not attending college/education or a day service for three or more days a week and had a score of 5 or more on the GDS‐LD (Cuthill *et al*. 2003). A score of 13 or more is suggestive of depression. A lower cut‐off score was used to improve access to the intervention to a wider group of participants. However, they still had some symptoms of depression to enable a change in scores to be detected as a result of the intervention.

Participants were excluded if they were unable to speak English or could not provide informed consent.

Volunteers were included if they were aged 18 or over and were available once a week for at least 1 hour, over 6 months. They were excluded if they had any previous criminal offences (documented on their Disclosure and Barring Service checks) or were unable to provide suitable references.

### Intervention and control groups

Participants in the intervention arm were matched to a volunteer based on shared interests and availability. The purpose of the befriending relationship was to provide emotional support and to facilitate access to activities in the community. The pairs had access to a resource booklet of local activities and were expected to meet once a week for about 1 hour, over 6 months, and to spend at least half the number of sessions in the community. Activities were recorded by the volunteer in a structured log‐book. Monthly supervision was provided to volunteers by the volunteer coordinator and was recorded on a supervision form. Participants with ID were also contacted regularly as part of the monitoring process. Volunteers were reimbursed their travel and additional expenses.

Participants in the control arm had access to the resource booklet. Both groups had access to usual care (including antidepressant medication). The majority of participants in the control arm were offered a befriender after completing follow‐up.

### Outcomes

Feasibility outcomes were assessed against our pre‐identified progression criteria, which were used to determine if a future large‐scale randomised controlled trial should be conducted. These included: recruitment rates of participants with ID and volunteers over 6 months (minimum target 35 participants); the number of matched pairs (target 70% to be matched); retention rate (less than 30% drop‐out rate at 6 months); adherence to the intervention, based on pairs completing a minimum of 10 meetings; acceptability of the intervention, based on interviews with participants with ID, volunteers, volunteer coordinators and carers. Adverse events and unintended consequences of the intervention were also recorded.

Outcome measures were assessed at baseline and 6 months follow‐up at a face‐to‐face assessment conducted by the research assistant. A telephone assessment was conducted where face to face contact was not possible (e.g. due to COVID‐19 restrictions).

The main clinical outcomes of interest in participants with ID were self‐reported symptoms of depression, measured by the GDS‐LD. Other outcome measures included self‐esteem, measured using the adapted Rosenberg Self‐esteem scale (Dagnan and Sandhu 1999); quality of life, assessed using the Maslow Assessment of Needs Scale‐Learning Disability (MANS‐LD; Skirrow and Perry 2009) and the adapted World Health Organisation Quality of Life questionnaire (WHO‐QOL‐8; World Health Organisation Quality of Life Assessment Group 1998); loneliness and social satisfaction, measured using the Modified Worker Loneliness Questionnaire (MWLQ; Stancliffe and Keane 2000); social support; measured using the Social Support Self Report for intellectually disabled adults (SSSR; Lunsky and Benson 1997) and social participation, measured using the Guernsey Community Participation and Leisure Assessment (GCPLA; Baker 2000).

Outcome measures in volunteers were self‐esteem, measured using the Rosenberg self‐esteem scale (Rosenberg 1965); psychological wellbeing, measured using the Warwick–Edinburgh Mental Wellbeing Scale (WEMWBS; Tennant *et al*. 2007); loneliness, measured using the UCLA loneliness scale (Russell and Peplau 1980) and attitudes towards people with ID, assessed using the Attitudes Towards Intellectual Disability Questionnaire (Morin *et al*. 2013).

The feasibility of conducting a health economic evaluation was assessed by collecting data on intervention costs from the befriending services; health services resource use, using the adapted Client Services Receipt Inventory (Beecham and Knapp 2001) by interviewing carers where possible; and health related quality of life, using the EuroQol‐ 5 Dimensions‐Youth (EQ‐5D‐Y) (Wille *et al*. 2010), and the EuroQol Visual Analogue Scale (EQ VAS), which were self‐reported by participants with ID.

### Impact of COVID‐19 pandemic

Only one participant did not complete the full 6 months of the befriending intervention prior to start of the COVID‐19 pandemic in March 2020. During the first national lockdown (March–May 2020), outstanding follow‐up assessments and all the qualitative interviews were conducted via telephone rather than face to face. The number of completed interviews was lower than expected, possibly due to the impact of the pandemic.

### Data analysis

Descriptive statistics were used to describe the whole sample and to compare both groups in relation to socio‐demographic characteristics and outcome measures. Due to the small sample size (refer to Recruitment section), an exploratory statistical analysis was undertaken only for the primary clinical outcome (depressive symptoms). The effect of befriending was estimated using a linear regression model with depressive symptoms at 6 months as the outcome and study arm as the main explanatory variable, adjusting for depressive symptoms at baseline.

## Results

### Recruitment, matching and retention

Recruitment was slower than anticipated. Twenty‐four referrals were received for participants with ID, of which 21 were assessed and 16 participants with ID were found to be eligible and agreed to be randomised. Twelve volunteers expressed an interest, and 10 completed the necessary checks and training. There were delays in setting up the study and an extension to the recruitment period was not granted by the funders. Consequently, significant changes to the recruitment strategy could not be implemented. Six out of the eight participants with ID in the befriending arm were matched to a befriender and met on at least one occasion. One participant could not be matched due to geographical distance and another because of concerns about risk. All the participants with ID completed their 6‐month assessment, but two volunteers dropped out (one because of conflict with the befriendee's paid carer at the supported living placement and another because the befriendee's mother was unhappy with the match). One participant was re‐matched after their volunteer left.

### Participant characteristics

The mean age of participants was 41.7 years; nine were female (56%) and eight (50%) were from non‐White ethnic backgrounds. The majority had a mild ID (81%), and the mean IQ was 55.3. Ten (63%) participants had a clinical diagnosis of depression, and nine (56%) were prescribed an anti‐depressant. Participants in the befriending arm (refer to Table 1) were older, mainly female and had fewer mental health comorbidities including depression. The mean GDS‐LD score was above 13 in both groups at baseline [18.6 (SD: 5.7) in the intervention arm and 21.4 (SD: 9.8) in the control arm] indicating that the majority of the participants met the threshold for a diagnosis of depressive disorder (refer to Table 2) and had scores well above 5 (minimum score for inclusion into the study).

**Table 1:** Demographics of participants with intellectual disabilities at baseline

	Control: numbers (%)	Befriending: numbers (%)
Age (years)—mean (SD)	34.4 (13.4)	48.9 (17.2)
Gender		
Male	5 (63%)	2 (25%)
Female	3 (38%)	6 (75%)
Ethnicity		
White	4 (50%)	4 (50%)
Other	4 (50%)	4 (50%)
Living arrangements		
Alone	1 (13%)	3 (38%)
With family	1 (13%)	1 (13%)
Supported living	5 (63%)	4 (50%)
Residential care	1 (13%)	0
IQ score—Mean (SD)	55.6 (10.3)	54.9 (4.1)
Degree of intellectual disability		
Mild	6 (75%)	7 (88%)
Moderate	2 (25%)	1 (13%)
Mental health diagnoses		
Depression	6 (75%)	4 (50%)
Psychosis/schizophrenia	1 (13%)	2 (25%)
Bipolar affective disorder	1 (13%)	0
Anxiety disorder	3 (38%)	2 (25%)
Autism	2 (25%)	0
ADHD	2 (25%)	0
Other	1 (13%)	1 (13%)
Taking antidepressants		
Yes	5 (63%)	6 (75%)
Comorbid epilepsy		
Yes	1 (13%)	0
Mobility		
Mobilises independently	8 (100%)	5 (63%)
Mobilises with walking stick or frame	0	3 (38%)

ADHD, attention‐deficit hyperactivity disorder; GDS‐LD, Glasgow Depression Scale for people with a Learning Disability; SD, standard deviation. Participants may have more than one mental health diagnoses.

**Table 2:** Outcomes in participants with intellectual disabilities at baseline and 6 months follow‐up

	Baseline assessment		6 month follow‐up	
	Control (*N* = 8)	Befriending (*N* = 8)	Control (*N* = 8)	Befriending (*N* = 8)
Depressive symptoms (GDS‐LD)	21.4 (9.8)	18.6 (5.7)	17.5 (6.5)	12.9 (6.7)
Self‐esteem (adapted Rosenberg self‐esteem scale)	20.8 (4.5)	22.4 (4.1)	21.4 (3.5)	23.9 (3.4)
Quality of life				
MANS‐LD	64.1 (10.3)	73.0 (9.4)	72.0 (5.5)	80.0 (9.9)
WHOQOL‐8	23.8 (4.7)	30.0 (4.4)	28.4 (3.5)	29.4 (5.3)
Loneliness and social satisfaction (MWLQ)				
Aloneness	7.6 (2.8)	8.4 (2.3)	7.0 (3.7)	8.6 (3.5)
Social dissatisfaction	6.8 (3.4)	7.9 (3.5)	8.3 (2.7)	7.6 (2.1)
Social support (SSSR) – median (IQR)				
Family	5.0 (0.5 to 6.5)	5.5 (2.5 to 6.5)	7.0 (4.0 to 9.0)	5.5 (1.5 to 7.0)
Staff	5.5 (5.0 to 7.0)	5.5 (3.5 to 7.5)	6.0 (5.0 to 8.0)	6.0 (2.5 to 7.5)
Friends	5.5 (3.5 to 7.5)	3.5 (1.5 to 6.0)	7.0 (4.0 to 9.0)	4.0 (3.0 to 6.5)
Partner	0 (0 to 7.5)	0 (0 to 3.5)	0 (0 to 0)	0 (0 to 5.0)
Social participation (GCPLA)	73.9 (25.9)	57.5 (23.4)	74.4 (20.4)	61.3 (22.5)

Statistics are mean (standard deviation) unless otherwise specified; GCPLA, Guernsey Community Participation and Leisure Assessment; GDS‐LD, Glasgow Depression Scale for people with a Learning Disability; MANS‐LD, Maslow Assessment of Needs Scale Learning Disability; MWQL, Modified Worker Loneliness Questionnaire; SSSR, Social Support Self‐Report for intellectually disabled adults; TAU, treatment as usual; WHOQOL‐8, Adapted World Health Organisation Quality of Life Measure. There was no missing data at baseline; there was 1 missing observation in the control arm (13%) for MWQL and SSSR at 6 months follow‐up.

The average age of volunteers was 33.2 years (SD:11.2). The majority were female (*n* = 70%), of White ethnicity (*n* = 7; 70%), employed (*n* = 7; 70%) and had qualifications equivalent to ‘A’ levels or above (*n* = 7, 70%). Four (40%) had prior experience of working with someone with ID.

### Adherence

The pairs met on average 11.8 times (range 1–21), which was above the minimum requirement of 10 meetings. However, only four out of the eight participants randomised to the befriending arm completed 10 or more meetings with a befriender. The average duration was 118 minutes. The majority of activities were outside the home (63.3%) and largely comprised visits to cafes (25.4%), restaurants (14.1%) and going for walks (16.9%). Having a conversation at home was the most frequent indoor activity (22.5%). Five volunteers participated in supervision and the mean number of sessions completed was 4.2. Common issues raised during supervision included managing the balance between work and family commitments with volunteering role, concerns about the participant's physical health and participant not having enough money to complete activities.

### Acceptability

Interviews were conducted with nine participants with ID (5 in the control arm and 4 in the intervention arm); five volunteers; three volunteer coordinators and four carers (3 were paid and one was a family member). The findings suggest that volunteers were satisfied with the trial procedures. However, some participants with ID reported difficulties understanding relevant information about the study, including randomisation. Participants, volunteers and carers reported positive experiences of the intervention, and there were benefits for all three groups. However, volunteers commented that they would have preferred more flexibility over the frequency of meetings and nature of contact (e.g. contacts via social media). Suggestions were made about improving the recruitment process for volunteers (e.g. through the hosting of events) and making the training more practical to enable volunteers to manage more challenging scenarios (such as the befriendee not having enough money during an outing).

### Adverse events/unintended consequences

There were no adverse events in participants with ID. One participant with ID was supported by their volunteer to raise a safe‐guarding alert after the participant revealed that they were being bullied by a member of staff, and the matter was investigated and resolved. One volunteer dropped out after they experienced distress following conflict with a carer at the befriendee's supported living placement.

### Analysis of outcome measures

The outcome measures at baseline and at 6 months for both participants and volunteers are displayed in Tables 2 and 3. Depressive symptoms were lower in the intervention group compared with the control group at 6 months [mean GDS‐LD scores of 12.9 (SD: 6.7) and 17.5 (SD: 6.5), respectively]. After adjustment for depressive symptoms at baseline, the GDS‐LD depressive symptoms score was four points lower in the intervention group compared to the control group (mean difference: −4.0; 95% confidence interval: −11.2 to 3.2). As expected for a pilot study, this was not statistically significant. This change is equivalent to an effect size of 0.5 standard deviations.

**Table 3:** Outcomes in befriending volunteers at baseline and follow‐up

	Baseline (*N* = 10)	Follow‐up (*N* = 8)
Self‐esteem (Rosenberg self‐esteem scale)	28.3 (2.3)	32.0 (4.6)
Psychological wellbeing (WEMWBS)	50.6 (6.9)	53.0 (6.0)
Loneliness (UCLA Loneliness Scale)	55.5 (3.9)	56.0 (4.0)
Attitudes (ATTID)		
Discomfort	30.5 (10.6)	29.9 (9.3)
Knowledge of capacity and rights	46.6 (6.6)	42.9 (6.9)
Interaction	26.6 (8.7)	24.4 (8.6)
Sensibility or tenderness	16.5 (2.9)	16.6 (3.5)
Knowledge of causes of intellectual disability	18.8 (6.4)	19.4 (7.0)

Statistics are mean (standard deviation) unless otherwise specified. ATTID, Attitudes Towards Intellectual Disability Questionnaire; UCLA, University of California, Los Angeles; WEMWBS, Warwick Edinburgh Mental Wellbeing Scale. There was 1 missing observation for the discomfort subscale of the ATTID.

### Exploratory heath economic evaluation

The total cost of the intervention was £5647.38, which includes the cost of recruitment (staff time), DBS and reference checks, the cost of staff time and resources to deliver training, supervision, monitoring calls and visits and volunteer expenses. The mean cost per participant was £701. It was possible to collect data on health resource use from carers in the majority of cases. However, there were inconsistencies between participants' ratings on EQ‐5D‐Y and EQ VAS scales, indicating that that participants may have found the EQ VAS measure difficult to understand.

## Discussion

This is the first randomised controlled trial of befriending in people with ID. We did not meet our recruitment target of at least 35 participants, which suggests that recruitment would be very challenging in a larger trial using the same recruitment strategy. The other feasibility outcomes relating to matching, retention of participants and adherence to the intervention were a little more promising but also warrant further attention due to issues with volunteers dropping out. The positive reports from volunteers and participants suggest that befriending was acceptable to those who participated. However, the low recruitment rate also indicates that it may not be widely acceptable to individuals with depressive symptoms. Based on our limited data, we found that depressive symptoms on the GDS‐LD in the befriending arm were four points lower at 6 months compared with the control arm, which is equivalent to a moderate effect size, but there were baseline differences in comorbidities between the two groups, and therefore, this finding should be interpreted with caution. One of the main mechanisms through which befriending may help to improve mood is behavioural activation. As the majority of meetings (over 60%) took place in the community, it suggests that befriending was helpful in promoting behavioural activation through engagement with community‐based activities.

One of the main challenges encountered in this trial were the impact of delays resulting from obtaining all the necessary approvals (e.g. sponsorship, ethics committee and local site approval) on participant recruitment and our ability to deliver the trial according to the protocol due to rigid timelines. These issues are common, with one study suggesting that the median time taken to open study sites is 9.7 months (Kearney *et al*. 2014). Although some efforts have been made by the Health Research Authority in England to reduce these delays in recent years (Ranieri *et al*. 2018), there is clearly a scope for improvement.

We identified three main challenges to participant recruitment. The first was the negative views of staff at befriending services towards randomisation, which may have affected their willingness to encourage individuals with ID to participate in a trial. Staff were acting as ‘gate keepers’, a common barrier to recruitment of participants with ID into randomised controlled trials (Oliver *et al*. 2002; Lennox *et al*. 2005); Second, the participants who were approached to take part in the study were not always willing to take part, possibly due to a lack of understanding or anxieties about taking part in a trial. Finally, lack of willingness to participate in the study may have been due to concerns about the befriending intervention itself, possibly because more individuals with severe symptoms of depression were approached than was anticipated (more than half had a clinical diagnosis of depression), who may have had lower levels of motivation to engage in befriending. Although participants with lower GDS‐LD scores were eligible to take part, these participants were not referred to the study, possibly because of confusion or misunderstanding about the inclusion criteria. Strategies to improve recruitment could focus on providing a clearer message to the referrers and more targeted recruitment of individuals with mild depressive symptoms. However, if individuals have fewer symptoms, then the changes in scores are likely to be less marked.

Although retention of participants was good, two volunteers dropped out of the study. More support should be provided to volunteers through social events and opportunities to meet other volunteers that enhance peer support. This may help volunteers to overcome challenges and sustain interest and engagement in their role. Effective communication between all the parties involved (befriending schemes, volunteers and carers) is paramount in ensuring that misunderstandings and conflicts between volunteers and carers are avoided.

A further challenge that we encountered was that one of the befriending services experienced financial difficulties, which had implications for staffing, and this is likely to have impacted on the recruitment of both volunteers and participants with ID. Inadequate financial resources and knowledge of appropriate research methods and outcome indicators are common barriers to conducting research with third sector organisations (Bach‐Mortensen and Montgomery 2018).

There were some limitations of the study. We were unable to implement changes to the recruitment strategy (such as opening up new recruitment sites and recruiting more befriending services), and to conduct the 12 month follow‐up due to delays in setting up the study. Consequently, the impact of relationships ending could not be explored fully, as this may be distressing for some individuals with ID (Heslop 2005). Our measures were predominantly self‐report, and it may have been useful to have included a proxy measure for depression and health related quality of life.

A future study should consider alternative recruitment strategies such as recruiting participants from multiple National Health Service trusts and from other sources (e.g. voluntary sector) and inviting more befriending services to take part in the study. Recruitment approaches could involve service users with ID being employed as researchers and recruiting participants (Hassiotis *et al*. 2020) by explaining the study to their peers and describing personal experiences of befriending and the impact it has had on them.

Future studies on befriending could have a broader eligibility criteria, with a focus on evaluating whether befriending leads to improvements in wellbeing and social outcomes. A further feasibility study that attempts to address some of the challenges encountered in this study may be helpful and a future full trial may be possible but alternative study designs, such as observational studies employing a longitudinal design, should be considered in evaluating the benefits of befriending.

## Source of funding

The study was funded by the Public Health Research Programme funding stream of the National Institute for Health Research (UK).

## Trial Registration

This trial is registered as ISRCTN63779614.

## Ethics approval

The study received ethical approval from the London‐City and East Research Ethics Committee (reference 18/LO/2188).

Permission to reproduce material from other sources: not applicable.

## Conflict of interest

There are no conflicts of interest.

## Data Availability

The data are available upon request. Please contact the corresponding author.
